# Determination of the Raman polarizability tensor in the optically anisotropic crystal potassium dihydrogen phosphate and its deuterated analog

**DOI:** 10.1038/s41598-020-73163-4

**Published:** 2020-10-01

**Authors:** T. Z. Kosc, H. Huang, T. J. Kessler, R. A. Negres, S. G. Demos

**Affiliations:** 1 Laboratory for Laser Energetics, University of Rochester, 250 East River Road, Rochester, NY 14623-1299 USA; 2 Lawrence Livermore National Laboratory, 7000 East Avenue, Livermore, CA 94550 USA

**Keywords:** Nonlinear optics, Characterization and analytical techniques, Raman spectroscopy

## Abstract

The Raman tensor of the dominant A_1_ modes of the nonlinear optical crystalline material potassium dihydrogen phosphate and its 70% deuterated analog have been ascertained. Challenges in determining the A_1_ mode tensor element values based on previous reports have been resolved using a specially designed experimental setup that makes use of spherical crystal samples. This novel experimental design enabled the determination of measurement artifacts, including polarization rotation of the pump and/or scattered light propagating through the sample and the contribution of additional overlapping phonon modes, which have hindered previous efforts. Results confirmed that the polarization tensor is diagonal, and matrix elements were determined with high accuracy.

## Introduction

Potassium dihydrogen phosphate (KDP) and its deuterated analog (DKDP) are widely used nonlinear optical materials. Because they can be grown in large sizes, these crystals are uniquely suited for large-aperture laser systems. KDP and DKDP plates are currently used for frequency conversion^1^, polarization control, and beam smoothing^2^ on inertial confinement fusion class lasers such as the NIF, LMJ, SG-III, and OMEGA^3−6^. However, transverse stimulated Raman scattering (TSRS) in large-aperture KDP/DKDP crystals limits their use at higher laser fluences by transferring energy to parasitic transverse beams, especially at shorter laser wavelengths^7,8^. Furthermore, the large dimensions of these optical elements, in combination with the high incident laser intensities, support the exponential increase of the TSRS signal that, if not properly managed, ultimately leads to damage for the optic and the surrounding hardware during laser operation^9^. To mitigate this risk, the TSRS must be modeled to determine suitable crystal cut configurations for design optimization. The model requires the Raman-gain coefficient, which is calculated from the propagation length (optic size), the laser intensity and pulse duration, and the spontaneous Raman scattering cross section^10,11^. The 3-dimensional description of the latter is based upon an accurate Raman polarizability tensor.

Although symmetry considerations provide guidance on the forms of the Raman tensors for a uniaxial crystal, group theory determines their exact form for all crystal classes^12^. KDP and DKDP, which are tetragonal crystals of the D_2d_ class, possess modes originating from symmetric lattice displacements both parallel and perpendicular to the optic axis (*c* axis in crystallography), resulting in type A_1_ (nondegenerate) and E (degenerate) modes, respectively. Asymmetric modes, B_1_ and B_2_, also exist for this crystal class. The Raman scattering spectra contain a number of distinct intense peaks at frequencies below about 1300 cm^–1^ arising from internal modes of the PO_4_ tetrahedron^13^ and numerous broad peaks above 1500 cm^–1^ arising from stretching and bending modes of OH bonds^14^. This work focuses on determining the Raman tensor for the symmetric A_1_ (915 cm^−^^1^) mode, which exhibits the highest cross section; therefore, it is the dominant mode in the generation of TSRS process. Although the Raman tensor for the A_1_ mode in KDP is expected to have only diagonal elements, a previous effort by Smith et al.^15^ detected Raman scattering in various experimental geometries, pointing to the existence of off-axis terms. Furthermore, their data suggested that two of those matrix elements have a dependence on the angle of the phonon wave vector with respect to the direction of the optic axis.

To the best of our knowledge, there is no published work that has attempted to resolve this apparent discrepancy. As a result, use of KDP and DKDP for high-power applications, and in particular for polarization control, has not been fully optimized, and there are currently no alternatives to these materials. More recently, the Raman scattering cross section has been measured using KDP and DKDP cubes^16^ in characteristic configurations that enabled estimation of the TSRS gain at relevant crystal cuts and excitation configurations using the empirical expression of the Raman tensor developed in Ref.^15^. Measurements using crystal cubes are limited to the crystal cut of the sample, thereby offering a very limited sampling for the Raman tensor behavior. To address this problem, initial experiments were performed using cylindrical samples cut with the optic axis (OA) at *θ* = 0° and 90° with respect to the axis of the cylinder (details in Supplementary Data S1), but these measurements did not resolve the ambiguity regarding the Raman tensor. Employing spherical samples, we obtain the necessary information to understand the exact form of the Raman tensor and the origin of the artifacts that gave rise to previous discrepancies.

The use of a spherical sample facilitates an ideal experimental system for the determination of the matrix elements of the Raman scattering tensor. This is because measurement of the Raman scatter cross section along distinct scattering geometries is directly related to specific tensor elements. Therefore, a single spherical sample can enable measurement of the Raman cross section at all scattering geometries required to extract the tensor elements. The details of the experimental system and methods for manufacturing the crystalline spherical samples were presented elsewhere^17^. This system enabled the ascertainment of the Raman polarizability tensor of the dominant A_1_ mode in KDP and DKDP by combining the experimental results with modeling for cross validation of the results. This approach also facilitated the identification of the origin of the measurement artifacts that have hindered previous efforts. We subsequently reevaluated the scattering cross-section value, since it was determined that the previous measurement^16^ may have also been affected by artifacts and therefore contained a systematic error in its value.

## Theoretical considerations

The Raman scattering cross section is proportional to $\left\{ {e_{{\text{p}}} ^{*} \cdot R \cdot e_{{\text{s}}} } \right\}^{2}$ where *e*_p_ and *e*_s_ are the unit electric polarization vectors of the pump and scattered light, respectively, and *R* is a 3 × 3 Raman polarizability tensor.

1 $$R = \left( {\begin{array}{*{20}c} {a_{xx} } & {a_{xy} } & {a_{xz} } \\ {a_{yx} } & {a_{yy} } & {a_{yz} } \\ {a_{zx} } & {a_{zy} } & {a_{zz} } \\ \end{array} } \right).$$

It follows that the value of tensor elements can be determined from measurements of the Raman signal in specific scattering geometries where the Raman cross-section depends only on one matrix element. KDP and DKDP belong to the tetragonal D_2d_ point group, and their Raman active vibrational symmetries and Raman tensors are well known^12^. Recent work continues to refine the formalism of Raman tensors for optically anisotropic crystals^18,19^. The most-intense Raman scattering line in KDP, which is therefore of concern for generating stimulated Raman scattering gain, is associated with the symmetric A_1_ mode. The theoretical description of the Raman tensor for the A_1_ mode is represented by a diagonal matrix. As one would expect from a uniaxial crystal, the *a*_*xx*_ and *a*_*yy*_ elements have the same value, *A*.

2 $$R = \left( {\begin{array}{*{20}c} A & 0 & 0 \\ 0 & A & 0 \\ 0 & 0 & B \\ \end{array} } \right).$$

The absence of off-diagonal matrix elements dictates that when both the pump and the scattered light are polarized and propagating along any one of the principal crystal axes—X, Y or Z (where the Z axis is the OA), the Raman scatter resulting from the A_1_ vibration will retain the same linear polarization state as the pump beam. Therefore, practically, the detected signal should be zero when the transmission axis of the analyzer is orthogonal to the pump polarization. However, Smith^15^, and even earlier Srivastava^20^, obtained results that suggested that off-axis terms exist for the A_1_ mode. Based on crystal symmetry, these potential off-axis elements are assigned as *C*, *D*, and *E*, and where *D* = *E,* because the crystallographic X and Y axes are indistinguishable in a uniaxial crystal.

3 $$R = \left( {\begin{array}{*{20}c} A & C & D \\ C & A & E \\ D & E & B \\ \end{array} } \right).$$

In this work, the laboratory coordinates are defined by lower-case italicized letters *x*, *y*, and *z*, while upper-case letters X, Y, and Z designate crystallographic axes. Standard Porto notation^21^ is used: *k*_p_ [*e*_p_ *e*_s_] *k*_s_ designates the propagation direction of the pump, *k*_p_, and scattered, *k*_s_, light as well as the unit electric polarization vectors of the pump, *e*_p_, and scattered, *e*_s_, light. The scattering geometry configurations that enable direct assessment of the matrix elements are provided in Table 1 (see columns 1 and 2) in the “Analysis and discussion” section. Due to the equivalency of the X and Y crystallographic axes, the 24 experimental configurations identified are reduced to 12 independent configurations in Table 1 (e.g., for the tensor element *A*, configurations Z[XX]Y and Y[XX]Z are equivalent to Z[YY]X and X[YY]Z, respectively). These configurations were used to extract the Raman tensor elements for KDP and 70% DKDP materials.

**Table 1:** Identification of the origin of the signal for the 12 independent Raman scattering geometries corresponding to individual Raman tensor elements in KDP.

Tensor element	Raman scattering geometry	Signal contributing modes				Normalizing scattering signal^a^	Comments on the 915-cm^−1^ signal Polarization rotation (PR) contribution increases with collection aperture size^b^
		915 cm^−1^	940 cm^−1^	970 cm^−1^	990 cm^−1^		
*A*^2^	Z[YY]X	**√**				1.00	Determine from maximum intensity value of trace
	X[YY]Z	**√**				1.00	
*B*^2^	Y[ZZ]X	**√**				0.62	Determine from average intensity value of trace
*C*^2^	Z[XY]X	Artifact			**√**	0.01	Strong PR
	X[YX]Z	Artifact			**√**	0.01	Strong PR
	Y[XY]X	Artifact			**√**	0.01	Very weak PR
*D*^2^	Z[XZ]X	Artifact	**√**			0.03	Weak PR
	Y[ZX]Z	Artifact	**√**			0.03	Weak PR
	Y[XZ]X	Artifact		**√**		0.02	Very weak PR
*E*^2^	Z[YZ]X	Artifact	**√**			0.02	Weak PR
	X[ZY]Z	Artifact	**√**			0.02	Weak PR
	Y[ZY]X	Artifact		**√**		0.02	Very weak PR

The modes that contribute to the scatter signal in the integration region of 860 to 960 cm−^1^ are explicitly called out. Data for *C, D,* and *E* elements [Eq. (3)] were acquired with ~ 1.0° collection half-angle. All signal values are normalized to the value of *A.*

^a^Polarization rotation artifacts from 915-cm^−1^ mode not included for *C*, *D*, *E* tensor element values.

^b^PR contribution to scattering signal can be properly excluded by fitting spectra to Lorentzian curves.

**Figure 1:**
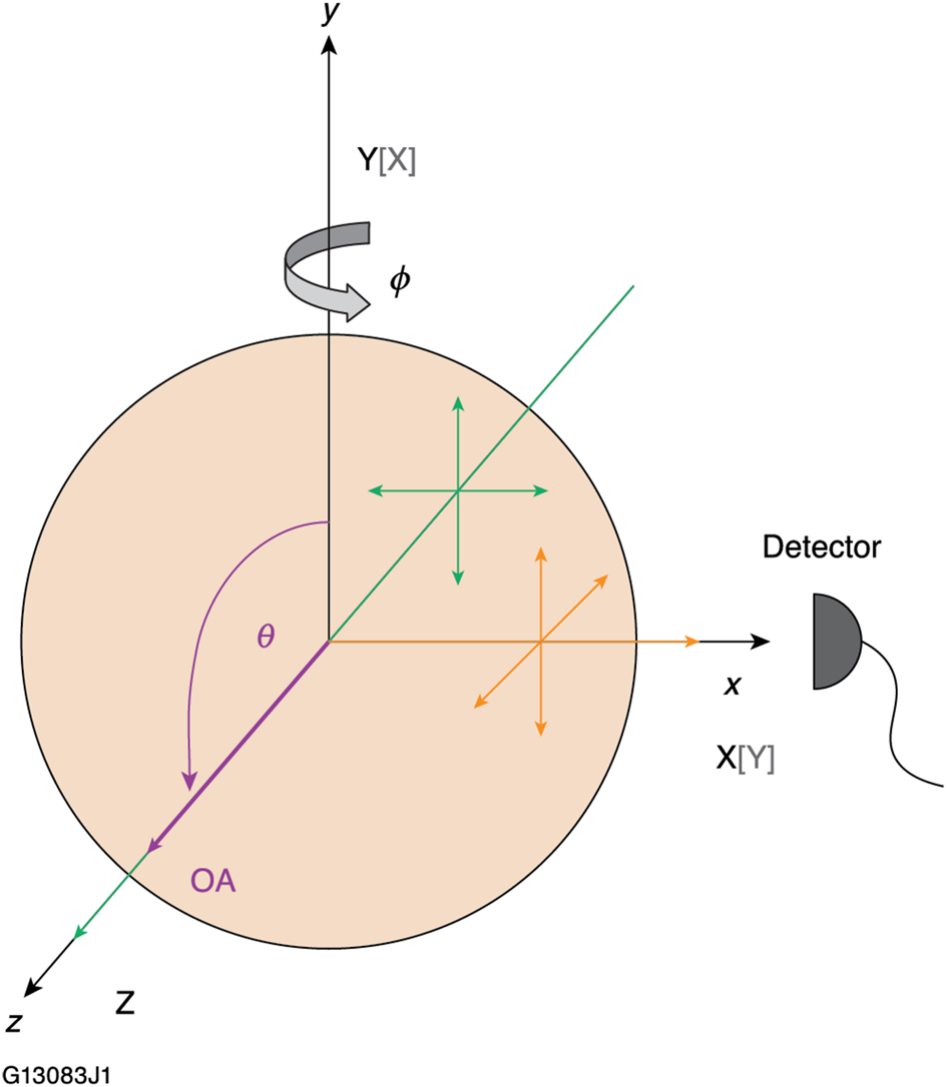
Experimental geometry for a Raman scattering measurement based on a spherical sample.

Using a spherical sample, “direct” tensor element measurements were acquired while rotating the sphere through 360° in the azimuthal plane, which is defined as the laboratory *x*–*z* plane and contains both the pump beam propagation and the Raman signal observation directions (Fig. 1). The angle *θ*  = 0° is defined along the laboratory *y* axis and is used to define the position of the OA. The azimuthal angle *ϕ* = 0° is defined along the laboratory *z* axis. For clarity, we further define the notation for each set of measurements while the sample is rotated (*ϕ*  = 0° to 360°) based on the initial configuration of the sample for *ϕ*  = 0° in reference to the crystal axis. Square brackets are omitted in the data set labels using the notation *k*_p_*e*_p_*e*_s_*k*_s_ to differentiate the notation of a data set and specific Raman scattering configurations found within each data set. For example, the Z[YY]X and X[YY]Z configurations appear at *ϕ*  = 0° and *ϕ*  = 90°, respectively, within the ZYYX data set.

## Results

**Figure 2:**
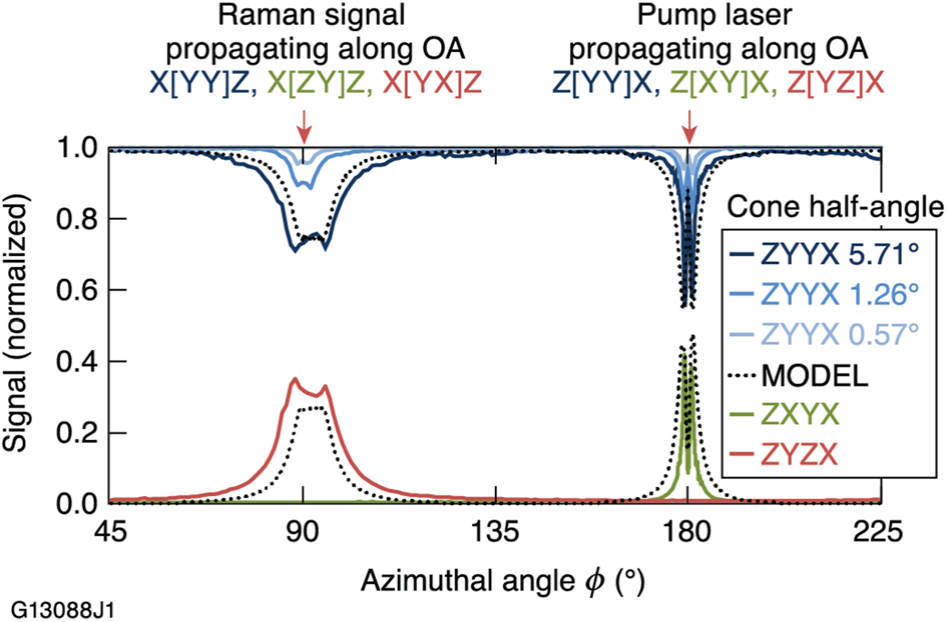
Data sets measuring the 915-cm^−^^1^ mode Raman scattering signal detected in 1° increments for sample rotation in the azimuthal plane are shown. The configurations used for tensor element determination are labeled above the azimuthal angle at which they are found. The model used a cone half-angle of 5.71°, which matches the half-angle of the collection aperture for the data set with multiple configurations.

Experimental results showing the signal of the A_1_ mode of KDP, centered at 915-cm^–1^ and integrated between 860 and 960 cm^–1^, are shown in Fig. 2. Data are acquired as a function of the azimuthal angle, *ϕ*, with the OA oriented in the azimuthal laboratory *x–z* plane. Each data point is obtained from a corresponding spectral profile. For readability, the graph in Fig. 2 presents data only between 45° and 225°. Complete data sets showing results for *ϕ*  = 0° to 360°, along with additional data sets acquired with (a) the X and Y crystallographic axes rotated 90° and (b) the OA aligned perpendicular to the azimuthal plane, are shown in Supplementary Data S2. For example, the trace labeled ZYYX in Fig. 2 provides data for scattering in the Z[YY]X configuration at azimuthal angles *ϕ*  = 0° or 180° (shown), while scattering data for the X[YY]Z configuration were collected at *ϕ*  = 90° (shown) or 270°. The polarization orientation of both the pump laser and the Raman scattering signal (and analyzer) for trace ZYYX are perpendicular to the azimuthal plane. Corresponding traces acquired when either (a) the analyzer or (b) the pump polarization is rotated by 90° (and parallel to the azimuthal plane) are shown as ZYZX and ZXYX, respectively.

Unexpected features such as double peaks and valleys are detected at the specific angles of significance for determining the matrix elements (see “Analysis and discussion” in next section). However, these features were reproduced by a coherent ray-tracing code implemented in Interactive Data Language (IDL) that does not consider any off-axis tensor elements, but takes into account that both the cone of focused pump rays and the scattering signal collection cone consist of an ensemble of rays at small angles with respect to the center of the cones (see dashed line profile in Fig. 2). The polarization states of these rays vary as each ray proceeds in the crystal due to birefringence effects. The effect is exacerbated when rays converge or diverge along the OA, because the differential phase between the ray components experiencing the ordinary and extraordinary indices of refraction is the greatest and leads to significant changes in the polarization state. Consequently, the Raman signal selectively collected with one linear polarization may in fact originate with the orthogonal polarization state in the region of the crystal where it is created. Porto^22,23^ and others^24,25^ discussed this phenomenon, to which they referred as “depolarization.” However, we chose to use the term “polarization rotation,” because the electric-field vector of each photon experiences polarization rotation due to the linear birefringence of the material, albeit to varying degrees depending on its path. We modeled the effect of converging (focused pump beam) and diverging beams (collected scattering) and how the cone angles affect the width and depth of the features. Three ZYYX data sets (Fig. 2, shaded blue traces) demonstrate the cone-angle dependence as the collection aperture diameter was varied between 1.5 mm and 15 mm corresponding to collection cone half-angles of 0.57° and 5.71°, respectively. A large signal collection angle allows for larger divergence of the collected signal from the propagation axis and thus increases the effect of polarization rotation. Modeling further suggests that while the width of the features is largely determined by the cone-angles, the sharpness and the position of the peaks and valleys is driven by the spectral resolution of they system (e.g. the slit width of the spectrometer).

**Figure 3:**
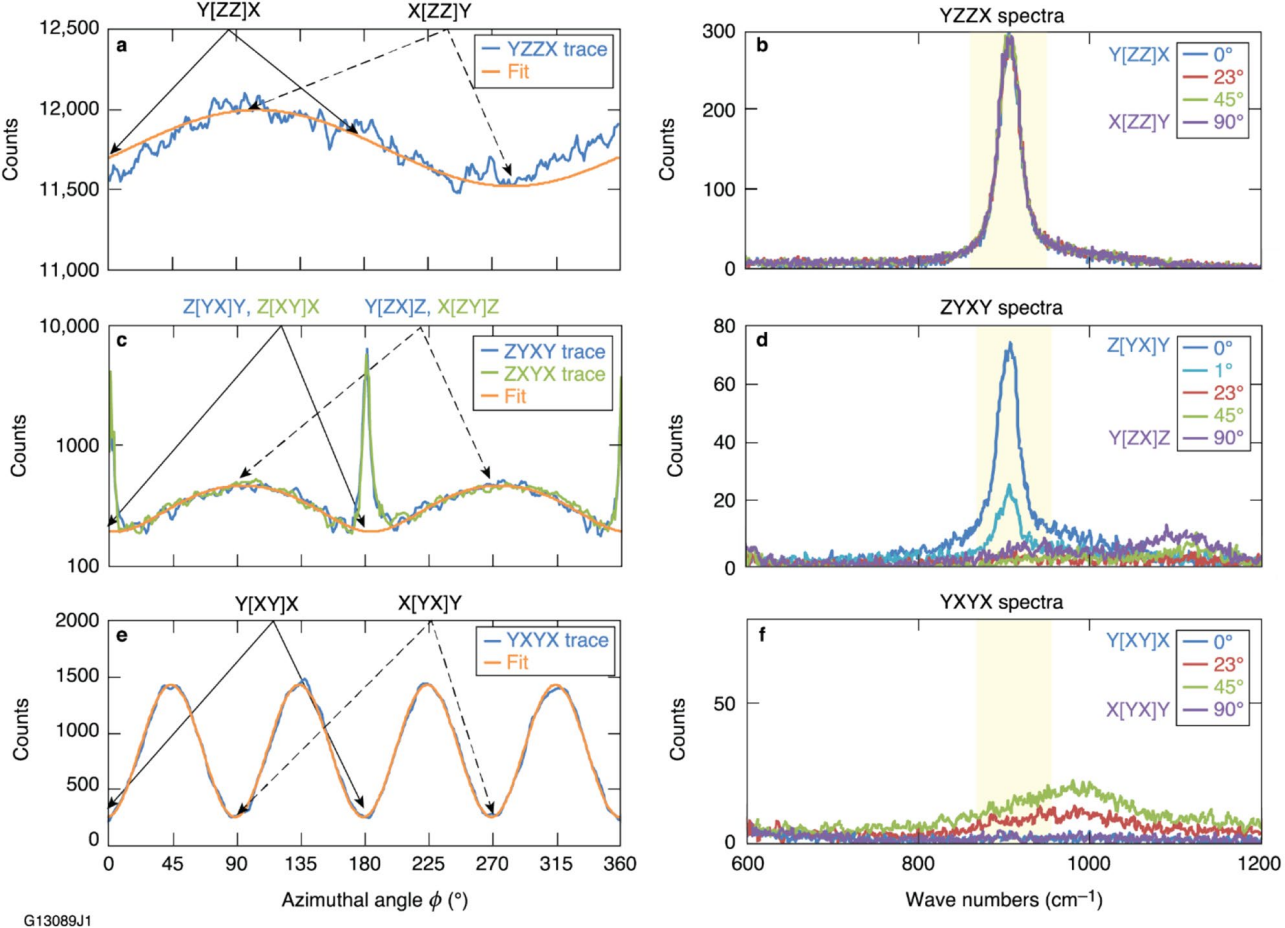
Selected traces and spectra demonstrate the presence of Raman scatter from (**a**,**b**) the dominant A_1_ mode of KDP, (**c**,**d**) polarization rotation, and (**e**,**f**) the overlap of neighboring modes. The outstanding reproducibility of orthogonal configurations is demonstrated in the ZYXY and ZXYX traces plotted in (**c**), using a semi-log plot. The 860- to 960-cm^−^^1^ integration region is shaded. Data in the traces are fit to a line or a sinusoidal curve to help quantify trends. Data acquired with a ~ 0.5° collection half-angle.

A closer examination of the Raman scattering spectral profile in the 860- to 960-cm^–1^ integration region for all spectra within each data set led to the identification of additional Raman modes whose signal partially overlaps into the wave-number range considered for the 915-cm^–1^ mode. Spectra for the YZZX data set (Fig. 3a), which includes both Y[ZZ]X and X[ZZ]Y configurations, shows a strong 915-cm^−^^1^ peak identical for all azimuthal positions (Fig. 3b). The situation is much different for the ZYXY trace (Fig. 3c), where strong peaks are visible at *ϕ*  = 0° or 180°. The equivalent trace acquired with the crystallographic X and Y axis swapped (ZXYX) is plotted to demonstrate the outstanding reproducibility of the data. Configurations probed in these data sets, Z[YX]Y and Y[ZX]Z (and their orthogonal analogues), correspond to data used for the determination of the *C* and the *D* matrix elements [see Eq. (3)], respectively. Therefore, the Raman peak at 915 cm^−^^1^ (A_1_ mode) should not be present. However, it is detected in the Z[YX]Y configuration due to polarization rotation effects that arise when the pump laser propagates along the crystal OA and produce sharp peaks at *ϕ*  = 0° or 180°. The large difference in the A_1_ mode peak magnitude (Fig. 3d) at *ϕ*  = 0° and 1° demonstrates the angular sensitivity of the polarization rotation effect. The spectra at *ϕ*  = 23°, 45°, and 90° reveal the presence of overlapping neighboring, low-intensity modes that produce the general sinusoidally varying scattering signal in these traces as a function of the azimuthal angle. Similarly, spectra corresponding to the YXYX trace (Fig. 3f) demonstrate the impact of adjacent modes whose scattering partially overlaps into the A_1_ mode spectral integration region and whose strength varies with the azimuthal angle *ϕ*.

**Figure 4:**
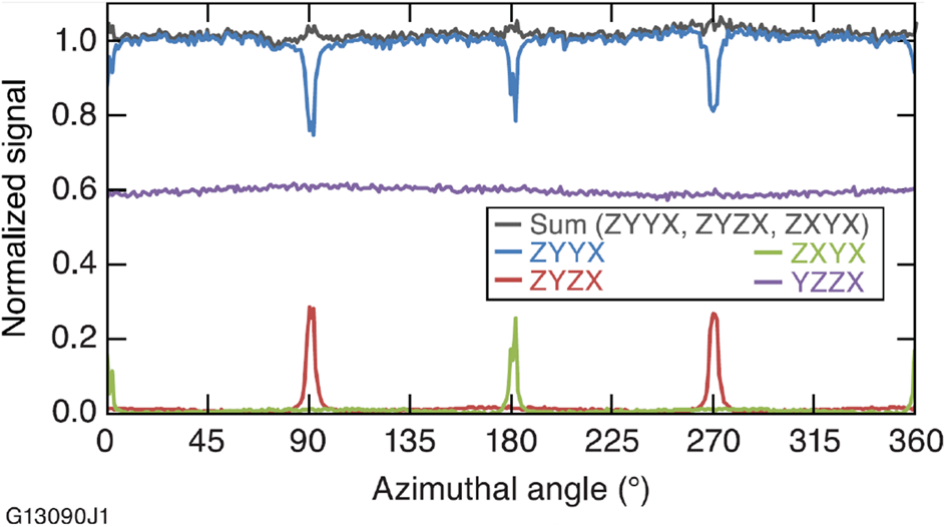
An approximately straight line (black) is produced when the three traces in which the pump laser and/or the scattering signal propagating along the optic axis experience polarization rotation are summed. The value of the *B* matrix element is determined from the YZZX trace (purple). Data was acquired with a ~ 1.0° collection half-angle.

Polarization rotation effects were most pronounced in configurations where either the pump beam or the scattered Raman signal propagated along the OA. This effect generated the peaks and valleys seen both in Fig. 2 and in an analogous data set acquired with a smaller (~ 1.0° collection half-angle) signal collection aperture shown in Fig. 4. In principle, a ZYYX trace (blue), acquired with the OA lying in the azimuthal plane, would be flat if the pump and signal rays all propagated parallel to the laboratory *z* and *x* axis, respectively. In practice, the pump beam is converging to a focus spot and the Raman scatter is collected with a half-cone angle as determined by the radius of the collection aperture. The polarization rotation effects observed at *ϕ*  = 0° and 180° (Z[YY]X configuration) occur as the pump beam propagates along the OA. The vertical polarization of the pump light is altered (i.e., a horizontally polarized component is produced), reducing the amount of Raman signal generated by vertically polarized pump light. An analogous condition exists at *ϕ*  = 90° and 270° (X[YY]Z configuration) where the Raman scatter signal propagates along the OA. In Fig. 4, the Raman scattering signal collection half-angle is only about twice the size of the pump beam focus half-angle (0.5°), and the full-width-at-half-maximum (FWHM) values of all polarization-rotation-produced features are the same to within 1°. In comparison, the scattering signal collection half-angle is 11× larger than the focusing half-angle for the data in Fig. 2, and the FWHM of features at 90° and 180° (governed by the smaller pump beam focus angle) vary by a factor of 5× to 6× .

The Raman scattering signal “lost” to polarization rotation effects described above appears in data sets in which there should be no Raman scattering from the A_1_ mode. Specifically, the wider peaks observed in ZYZX traces at *ϕ*  = 90° and 270° (X[YX]Z configuration with Raman scattering propagation along the crystal OA, vertically polarized pump beam and horizontally positioned analyzer) correspond to the signal of the X[YY]Z configuration that underwent polarization rotation. As a result, the XYYZ signal exhibits valleys in the Raman intensity at the same angles (*ϕ*  = 90°, 270°) where the ZYZX trace (red) exhibits peaks. Similarly, polarization rotation of the pump light leads to valleys in ZYYX traces at *ϕ*  = 0° and 180° (Z[YY]X configuration). The corresponding “lost” signal appears as sharp narrow peaks in the ZXYX trace (green) at the same angles (Z[XY]X configuration). Here the polarization rotation of the pump light generates a complementary Z[YY]X component that gives rise to the observed signal where no signal should be present. If all light affected by polarization has been entirely detected in these configurations, then the sum of the scattering signal of these three traces should be the same at every azimuthal angle. Figure 4 shows that the sum of the traces, normalized to the peak intensity of trace ZYYX, is essentially a straight line (black trace).

## Analysis and discussion

Raman scattering from the 915-cm^−^^1^ A_1_ mode of KDP should only be present in configurations corresponding to the diagonal matrix elements *A* and *B.* Nonetheless, Raman scattering was detected in the relevant signal integration region for all configurations, either arising from polarization rotation effects or due to scattering from neighboring modes (Table 1). Spectra for configurations determining the *A* and *B* elements show a strong 915-cm^−^^1^ peak with a broad shoulder on the red-shifted side (Fig. 3b). Polarization rotation produces the strong, unexpected peaks in the configurations that correspond to the matrix element *C* and in which the pump beam or the scattering signal propagates along the optic axis (Z[YX]Y, X[YX]Z, Z[XY]X, Y[XY]Z).

As shown in Fig. 3d, the 915-cm^−^^1^ peak and its shoulder disappear quickly as the sample sphere is rotated away from the Z[YX]Y configuration, while the signal of an additional Raman mode peaked at ~ 940 cm^−^^1^ increases and reaches a maximum strength at azimuthal angles 90° and 270°, corresponding to the Y[ZX]Z configuration (tensor element *D*).

The conditions for polarization rotation of the 915-cm^−^^1^ mode do not exist for the remaining configurations that determine the tensor element *C,* X[YX]Y and Y[XY]X, because the OA is aligned vertically. Figure 3e shows that the YXYX trace has a four-cycle sinusoidally varying Raman signal, and spectral analysis determines that it arises from the tail of a mode peaked at 990 cm^−^^1^. To determine the correct magnitude of scatter from the 990-cm^−^^1^ mode to be assigned to the matrix element *C*, the sample sphere is meticulously aligned to position the crystallographic Y axis along the laboratory *z* axis, which occurs when the sine curve minimum is found at *ϕ* = 0° (Ref. 17). For all configurations determining off-axis elements, particularly those in which the pump beam or the collection signal propagates along the OA, weak 915-cm^−^^1^ mode artifacts arise from polarization rotation and contribute to the increasing scattering signal as the collection half-angle increases.

Both small- and large-aperture experiments, which either reduced polarization rotation artifacts or improved signal-to-noise ratios, respectively, were important for the identification of neighboring Raman modes. A review of spectra for all experimental configurations confirmed that the Raman tensor for the dominant A_1_ mode is diagonal and contains *no* off-axis terms. The Raman scattering spectra suggest that nonzero signals corresponding to off-axis tensor elements arise from additional modes whose scattering partially overlaps with the 915-cm^−^^1^ mode integration region. The difficulty in reproducing values for the *A* and *B* tensor elements in past experiments was largely due to the strong polarization rotation effects, which are dependent upon experimental conditions (such as the focus and the collection angles). The previous assignment of an empirically determined angle-dependent tensor element *C* could have resulted from polarization rotation effects and/or the angle *ϕ* -dependent scattering from the 990-cm^−^^1^ mode. Similarly, scatter from neighboring modes with peaks at 940 cm^–1^ and 970 cm^–1^ led to nonzero signal values corresponding to tensor elements *D* and *E*. Since the neighboring modes are not the focus of this work, they will not be discussed further in this paper.

The primary goal for this work is to understand the Raman tensor for the dominant A_1_ mode and determine the value of the matrix elements. The Raman scattering signal should be maximum in the Z[YY]X geometry (or in the equivalent geometries) corresponding to the value of *A*^2^ (of matrix element *A*). In the following analysis, all other tensor elements are normalized to the value of *A*, which is assigned the maximum value of trace ZYYX (or ZXXY). The matrix element *B* can be determined by averaging of the entire trace YZZX. The A_1_-mode scattering in the YZZX trace does not experience polarization rotation, and modeling has confirmed that the mild oscillation seen in the YZZX trace in Figs. 3a and 4 is attributed to a slight misalignment of the sphere (within the resolution of our system to control the sphere position). Polarization rotation, however, affects the ZYYX measurement, and experiments confirmed that the impact of polarization rotation on the ratio of the matrix elements *B*/*A* increases with an increasing signal collection aperture size. A series of experiments was performed to further explore the dependence of the ratio of matrix elements *B*/*A* at various azimuthal angles *ϕ*. The results indicated that for angles *ϕ*  = 44°–46°, geometries for which polarization rotation is minimized in the ZYYX trace, the *B*/*A* ratio value is essentially constant as a function of aperture size. A power fit was used to extrapolate both matrix element values *A* and *B* as the aperture size approaches zero, thereby determining an accurate value for the ratio of *B*/*A*. The normalized tensors for the A_1_ mode of KDP and 70% DKDP, analyzed in the same manner, are provided in Eq. (4).

4 $$R_{{{\text{KDP}}}} = \left( {\begin{array}{*{20}c} 1 & 0 & 0 \\ 0 & 1 & 0 \\ 0 & 0 & {0.79 \pm 0.01} \\ \end{array} } \right){\text{ and }}R_{{70\% {\text{ DKDP}}}} = \left( {\begin{array}{*{20}c} 1 & 0 & 0 \\ 0 & 1 & 0 \\ 0 & 0 & {0.76 \pm 0.02} \\ \end{array} } \right).$$

For the most-accurate modeling of TSRS effects in large KDP/DKDP optics, the contribution of the neighboring modes to the Raman scattering signal may need to be considered. Although these adjacent modes have much lower cross sections, they are strong enough to have contributed, in addition to the effects of polarization rotation, to the mischaracterization of the Raman tensor with the inclusion of off-axis matrix elements.

While the Raman tensor interrelates the electric-field vector of the excitation and the Raman scattering radiation, additional factors affect the absolute value of the Raman scattering cross section with most important being the wavelength (Raman scattering intensity is proportional to *λ*^−4^, where *λ* is the laser wavelength). Previous efforts^16^ to quantify the Raman scattering cross section in KDP and DKDP crystals at three different wavelengths important for high-power laser applications (266 nm, 355 nm, and 532 nm) involved a relative measurement of the signal using cubic samples in the scattering geometry that corresponds to the *A*^2^ value of the tensor elements scaled to the signal from a source with a known cross-section value—water. For the purposes of this experiment, a cubic sample and a cell containing de-ionized water were used, essentially duplicating the experimental procedure used in Ref. 16. The results indicated that there is very small (of the order of 1% or less) depolarization in the Z[XX]Y geometry used in Ref. 16 when using a cube sample and a long-focal-length lens to focus the excitation light into the sample (these values are significantly higher in the sphere samples due to higher focusing power of the pump laser light by the sphere). Recall there is no depolarization in the X[ZZ]Y geometry. We therefore conclude that the Raman cross-section measurements presented in Ref. 16 are free from any inherent systematic error by artifacts. We used the same method to recalculate the Raman cross section in KDP using a small collection angle which is more relevant to practical implementations of TSRS. The Raman cross section of water (acquired in the same orthogonal signal collection geometry) was used as the reference material. The Raman cross section of water was recently determined with more accuracy to be $\left( {{{d\sigma_{{{\text{H}}_{2} {\text{O}}}} } \mathord{\left/ {\vphantom {{d\sigma_{{{\text{H}}_{2} {\text{O}}}} } {d\Omega }}} \right. \kern-\nulldelimiterspace} {d\Omega }}} \right)_{{90^{ \circ } }} = 5.74\;\, \times \;\,10^{ - 30} {{{\text{cm}}^{2} } \mathord{\left/ {\vphantom {{{\text{cm}}^{2} } {{\text{sr}}}}} \right. \kern-\nulldelimiterspace} {{\text{sr}}}}$ (Ref. 26). The following approach^16,27^ was used to calculate the Raman scattering cross section of KDP using the Raman scattering cross section of water for normalization

5 $$\left( {\frac{{{\text{d}}\sigma_{{{\text{KDP}}}} }}{{{\text{d}}\Omega }}} \right)_{90^{ \circ }} = \left( {\frac{{F_{{{\text{KDP}}}} }}{{F_{{{\text{H}}_{{2}} {\text{O}}}} }}} \right)\left( {\frac{{n_{{{\text{KDP}}}} }}{{n_{{{\text{H}}_{{2}} {\text{O}}}} }}} \right)^{2} \left( {\frac{{1 - r_{{{\text{H}}_{{2}} {\text{O}}}} }}{{1 - r_{{{\text{KDP}}}} }}} \right)\left( {\frac{{M_{{{\text{H}}_{{2}} {\text{O}}}} }}{{M_{{{\text{KDP}}}} }}} \right)\left( {\frac{{1 + \rho_{{{\text{KDP}}}} }}{{1 + \rho_{{{\text{H}}_{{2}} {\text{O}}}} }}} \right)\left( {\frac{{{\text{d}}\sigma_{{{\text{H}}_{{2}} {\text{O}}}} }}{{{\text{d}}\Omega }}} \right)_{{90^{ \circ } }} ,$$

where *F* is the measured signal (corrected for instrument response, *n* is the index of refraction, *r* is reflection arising from an air material interface, *M* is the molecular density, and *ρ* is typically referred to as the depolarization intensity ratio. The depolarization factor of water was remeasured and found to be $\rho_{{{\text{H}}_{2} {\text{O}}}} = 0.17,$ which matched the literature value in Ref.^16^. For the determination of the cross section of the specific A_1_ KDP Raman mode, we used *ρ*_KDP_ = 0. The molecular densities at room temperature are: *M*_KDP_ = 1.0322 × 10^22^ molecules/cm^3^ (as provided by the manufacturer, Cleveland Crystals, Inc.) and $M_{{{\text{H}}_{{2}} {\text{O}}}} = 3.335\;\, \times \;\,10^{22}$ molecules/cm^3^. Given these parameters, the Raman cross-section for KDP was determined to be $\left( {{{{\text{d}}\sigma_{{{\text{KDP}}}} } \mathord{\left/ {\vphantom {{{\text{d}}\sigma_{{{\text{KDP}}}} } {{\text{d}}\Omega }}} \right. \kern-\nulldelimiterspace} {{\text{d}}\Omega }}} \right)_{90^\circ } = 6.45 \pm 0.25 \times 10^{ - 30} \;{{{\text{cm}}^{2} } \mathord{\left/ {\vphantom {{{\text{cm}}^{2} } {{\text{sr}}}}} \right. \kern-\nulldelimiterspace} {{\text{sr}}}}.$ This value is about 20% lower to that presented in Ref. 16, which is nonetheless within the stated estimated experimental error. Part of the difference may arise from the different collection angle used in each experiment, which was with ~ 1.0° and ~ 20.0° collection half-angle in the present work and in Ref.^16^, respectively. However, due to the use of water as reference (which involves normalization of the intensity of Raman lines emitting at different wavelengths with separation of about 90 nm), the spectral calibration of the detection system is of critical importance. Three different Tungsten-Halogen commercially available calibration lamps were used in this work; ThorLabs SLS201L, Ocean Optics HAL2000 and, Ocean Optics HL-3P–CAL. Each of these lamps provided a different value, although the value of the latter two was very similar. We chose to use the data of the third lamp, which was newly calibrated by the manufacturer. Lamp positioning and alignment during calibration also contributed to variation in the estimated value. The value given above is the average of six different measurements. An approximation for the Raman cross section of the neighboring modes noted in Table 1 can be determined by scaling the value of the A_1_ mode cross section to the value of the normalized scattering signal for each mode. Using the Raman scattering spectrum from KDP (or DKDP) one can also extract an approximation for the scattering cross section of the other modes (by normalizing their intensity to that of the A_1_ mode). However, depolarization artifacts and spectral overlapping between modes would require a more thorough analysis of the Raman tensor for each mode. This analysis is outside the scope of the present article and will be provided in future work.

## Methods

An experimental system using spherical samples was designed to enable Raman scattering measurements while rotating the sample to enable a more-accurate assessment of the tensor elements. A detailed description of this experimental system has been presented elsewhere^17^. Sphere size for the KDP and DKDP samples ranged between 30 and 32 mm in diameter. The signal of the A_1_ mode of KDP, centered at 915 cm^–1^, was integrated between 860 and 960 cm^–1^. The 70% DKDP signal was integrated between 810 and 920 cm^−^^1^. All spectra were saved for further analysis. Measurements using a KDP spherical samples were complemented by analogous measurements using cubic samples. Data were acquired using a 532-nm (GEM, LaserQuantum) pump laser at 95 mW and a Horiba iHR320 spectrometer (100-μm slit, 1200-ln/mm grating). Raman peaks were fit using Lorentzian curves. The polarization and spectral response sensitivity of the detection arm of the setup (primarily the spectrometer and detector system) were determined using a Tungsten-Halogen lamp (Ocean Optics, HL-3P-CAL) to normalize the data.

The Raman scattering signal of de-ionized water was measured using the same experimental setup used for all KDP measurements. The water was held in a 1-cm fused-silica cuvette (Starna Cells Inc.) whose position was well-registered on the sample holder. A Z-cut KDP cube was placed in the exact same location, and the Z[YY]X configuration was used to collect the Raman scattering signal.

## Conclusion

A specially designed experimental setup enabled enhanced measurements and an improved understanding of the Raman scattering signal in KDP and DKDP crystals. Discrepancies in tensor element values in earlier investigations arose from: (1) polarization rotation of the pump and/or scattered light propagating through the sample and (2) overlapping signal from neighboring Raman modes. The polarization tensor elements for the A_1_ Raman mode have been accurately determined for KDP and 70% DKDP, and the salient features in the experimental spectra were well reproduced by ray-trace modeling, thereby enabling a more-accurate evaluation of the TSRS gain and optimization of polarization rotator design in future work. Future experiments will continue to explore both polarization rotation effects and the contribution of additional (to A_1_) modes in the Raman scattering region of interest. Finally, the Raman scattering cross section for the A_1_ Raman mode of KDP under 532-nm excitation has been determined to be $\left( {{{{\text{d}}\sigma_{{{\text{KDP}}}} } \mathord{\left/ {\vphantom {{{\text{d}}\sigma_{{{\text{KDP}}}} } {{\text{d}}\Omega }}} \right. \kern-\nulldelimiterspace} {{\text{d}}\Omega }}} \right)_{90^\circ } = 6.45 \times 10^{ - 30} \;{{{\text{cm}}^{2} } \mathord{\left/ {\vphantom {{{\text{cm}}^{2} } {{\text{sr}}}}} \right. \kern-\nulldelimiterspace} {{\text{sr}}}}.$

## Supplementary information

Supplementary file 1.
