# Supplementary material for: Determination of the Raman polarizability tensor in the optically anisotropic crystal potassium dihydrogen phosphate and its deuterated analog
Source: Sci Rep. 2020 Oct 1;10:16283. doi: 10.1038/s41598-020-73163-4 (PMC7529753; doi:10.1038/s41598-020-73163-4)
Supplement: Supplementary file 1 [file 41598_2020_73163_MOESM1_ESM.pdf]

Determination of the Raman Polarizability Tensor in the Optically Anisotropic Crystal Potassium Dihydrogen Phosphate and its Deuterated Analog

T. Z. Kosc[1,a)], H. Huang[1], T. J. Kessler[1], R. A. Negres[2], and S. G. Demos[1]

[1] *Laboratory for Laser Energetics, University of Rochester, 250 East River Road, Rochester, NY 14623–1299 USA*

[2]*Lawrence Livermore National Laboratory, 7000 East Avenue, Livermore, CA 94550 USA*

[a)] *Author to whom correspondence should be addressed: tkos@lle.rochester.edu.*

Supplementary Data S1: Preliminary experiments performed using cylindrical samples

The first set of experiments was performed using cylindrical samples. Such samples still offer the ability to rotate along a specific axis, but different samples are required for each crystal cut configuration. Four cylinder-shaped KDP samples were cut and polished from KDP plates with a different phase-matching angle, θ, between the cylinder axis and the principal Z axis of the crystal (optic axis): Sample 1 with $\theta = 0°$, Sample 2 with $\theta = 90°$ Sample 3 with $\theta = 59°$ and Sample 4 with $\theta = 41°$. Directly relevant to this work are samples 1 and 2. Results from Samples 3 and 4 will be discussed in future publications. The final dimensions (height × diameter) of the cylinder samples were ~1 cm × 1 cm and 2.7 cm × 0.9 cm for Samples 1 and 2, respectively. The path length through the samples (i.e., radius) had to be kept to a minimum in order to reduce the birefringence effects, leading to a vertical displacement of the Raman signal on the charge-

coupled–device (CCD) camera upon rotation of the samples. To better mitigate this problem, the Raman signal was integrated along this direction. Due to technical difficulties encountered in the fabrication and polishing processes of the cylinder-shaped samples, the final surfaces exhibited some degree of curvature variations and roughness, leading to excessive Rayleigh scattering. At those particular locations, the measurement error was reduced by translating the samples along the y axis and averaging a series of measurements at various heights (for the same rotation angle).

The experimental arrangement, with a geometry similar to the one shown in Fig. 1, consisted of a continuous wave (cw) Ar ion laser operating at 363.8 nm with 200 mW of power, UV-grade fused-silica lenses (L) for focusing of the pump beam in the bulk of the sample (f = 10 cm) and collecting the Raman-scattered light, and a SPEX imaging spectrometer (Jobin Yvon, SPEX 320) equipped with a liquid-nitrogen–cooled CCD camera (Princeton Instruments) for acquisition of Raman spectra. A polarizer (before the sample) and an analyzer in front of the spectrometer entrance slit were used to control polarization. The cylinder sample axis was aligned along the laboratory y axis and rotated 360° (by the angle ϕ, in 10° increments) in the laboratory x–z (azimuthal) plane. Figure S1 shows the integrated Raman scattering intensity for all four polarization/analyzer combinations for both cylinders.

The unique features observed in several traces and which have now been determined to arise due to polarization rotation effects, were first observed in the cylindrical samples [Fig. S1].

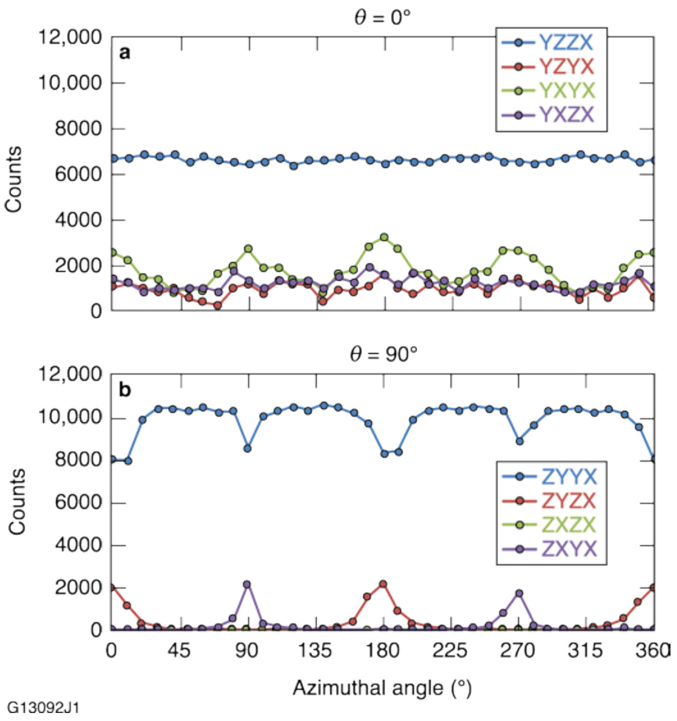

Figure S1. Raman scattering intensity at 915 cm^{-1} for $\theta = 0°$ and 90° cut KDP cylinders as a function of the azimuthal rotation angle ϕ. (a) Note that the trace YZZX is plotted on the same scale as the traces in (b) while the other three traces (YZYX, YXYX, and YXZX) are plotted on a secondary axis, with a scale an order of magnitude lower, to show the detail of the data.

The peaks and valleys produced when either the pump laser or the scattering signal propagate along the crystal optic axis are visible for the ZYYX, ZYZX, and ZXYX traces. Likewise, the YZZX trace is nearly flat, and the four-cycle sine pattern in signal intensity for trace YXYX is easily recognized. The tensor element *B*/*A* ratio (obtained using the analysis adapted for the spherical samples) is roughly of the order of ~0.8, which is within 2% of the value calculated above. These results demonstrated that the use of cylindrical sample still provides only limited information. They highlighted the need for high-quality polishing of the samples and data acquisition at very small increments to better resolve the observed features.

Supplementary Data S2: Results from all independent configurations

As discussed in the manuscript and shown in Table 1, there are 12 independent scattering configurations in the rotation of the sphere around on the crystal axis. Four of the configurations can be considered duplicates due to the symmetry of a uniaxial crystal like KDP where the crystal X and Y axes are indistinguishable. Results from three configurations and their spectra are provided in Fig. 3 of the manuscript. The detailed results of the additional five configurations are provided in Fig. S2. Raman spectra acquired at azimuthal angles $\theta = 0°$, 23°, 45°, and 90°, shown for each configuration, demonstrate how the Raman scattering spectra within the 860- to 960-cm^{-1} integration range change. The spectra do not change in configurations used to determine the tensor element *A*, *B*, but vary significantly for the element *C*, *D*, and *E* configurations.

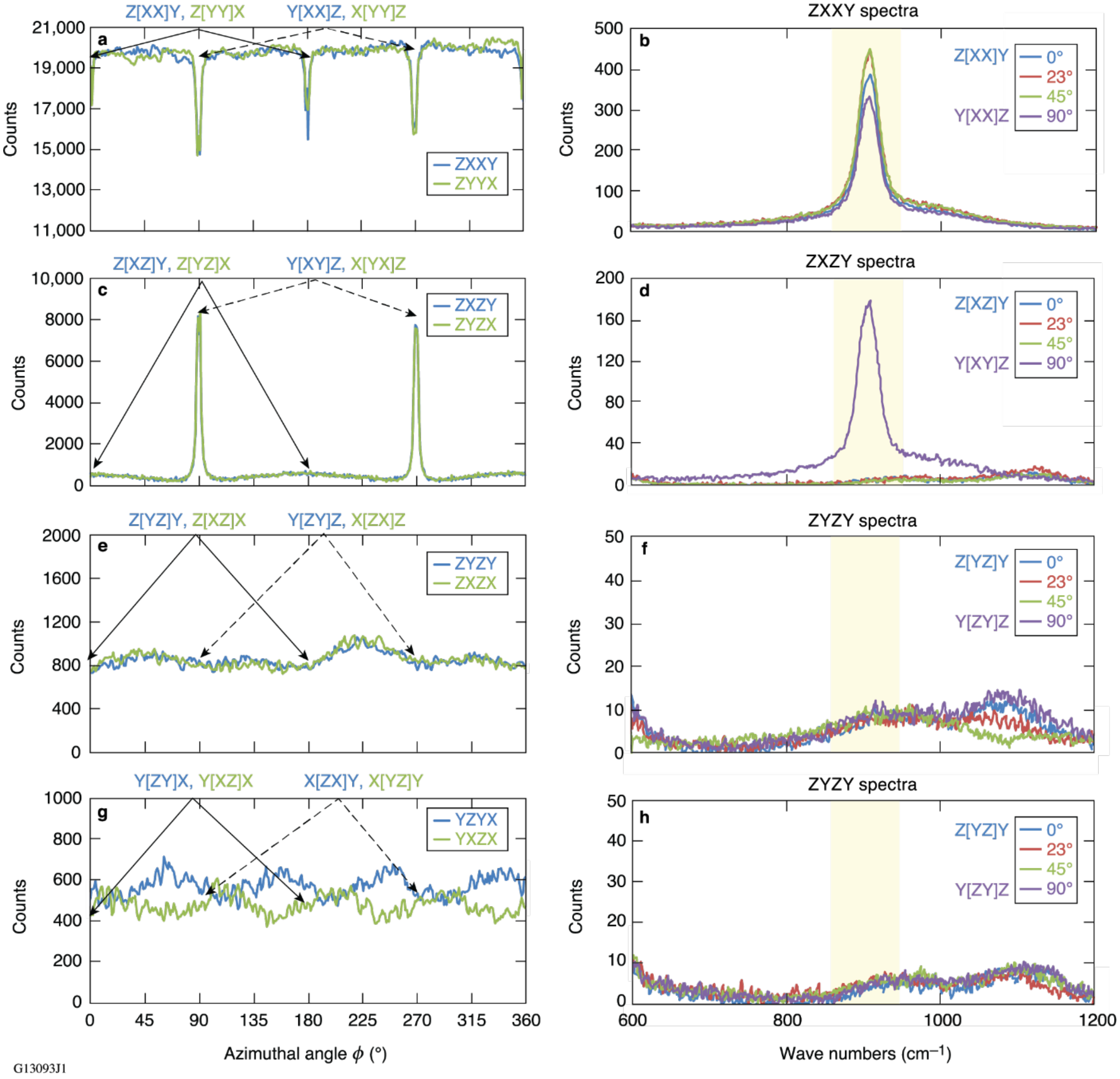

Figure S2. The intensity of the 915-cm^{-1} mode as a function of the azimuthal angle (right) and selected Raman scattering spectra (left) for different scattering configurations: [(a),(b)] ZXXY and ZYYX, [(c),(d)] ZXZY and ZYZX, [(e),(f)] ZYZY and ZXZX, and [(g),(h)] YZYX and YXZX. The data were acquired with a ~0.5° collection half-angle.

Figures S2(a) and S2(b) show that the dips in the 915-cm^{-1} mode strength for ZXXY trace in the graph are a consequence of polarization rotation. The peak intensities of the spectra at $\phi =$

0° and 90° are lower than those detected at ϕ = 23° and 45°. In Figs. S2(c) and S2(d), the strong 915-cm^{-1} peak at ϕ = 90° and 270° (Y[XY]Z configuration) is due to polarization rotation effect. The small, gently varying signal in between the 915-cm^{-1} peaks is due to scattering from neighboring Raman modes at 940 cm^{-1} and 970 cm^{-1}. The Raman spectra in Figs. S2(e) and S2(f) indicate that the Raman signal detected in these configurations is due to the tails of modes peaked at ~940 cm^{-1} and 970 cm^{-1}. Similarly, the Raman spectra in Figs. S2(g) and S2(f) involve only the 940-cm^{-1} and 970-cm^{-1} modes. Note that the signal in the two traces have the opposite phase determined by the direction of the pump and signal polarizations.
